# Impact of Kawasaki disease on juvenile idiopathic arthritis in real-world patients: A population-based cohort study

**DOI:** 10.3389/fimmu.2022.1025553

**Published:** 2022-12-08

**Authors:** Li-Chin Liao, Yi-Hsiu Fu, Chieh-Mao Chuang, Pei-Lun Liao, James Cheng-Chung Wei, Yun-Ching Fu

**Affiliations:** ^1^ Department of Pediatrics, Wuri Lin Shin Hospital, Taichung, Taiwan; ^2^ Department of Pediatrics, Childrens Medical Center, Taichung Veterans General Hospital, Taichung, Taiwan; ^3^ Department of Medical Research, Chung Shan Medical University Hospital, Taichung, Taiwan; ^4^ Department of Allergy, Immunology & Rheumatology, Chung Shan Medical University Hospital, Taichung, Taiwan; ^5^ Institute of Medicine, Chung Shan Medical University, Taichung, Taiwan; ^6^ Graduate Institute of Integrated Medicine, China Medical University, Taichung, Taiwan; ^7^ Department of Pediatrics, School of Medicine, National Chung Hsing University, Taichung, Taiwan; ^8^ Department of Pediatrics and Institute of Clinical Medicine, National Yang Ming Chiao Tung University, Taipei, Taiwan

**Keywords:** Kawasaki disease, juvenile idiopathic arthritis, longitudinal health insurance database, interleukin-1β, interleukin-6

## Abstract

**Objectives:**

Recent research has demonstrated the commonality of several biological markers between Kawasaki disease (KD) and juvenile idiopathic arthritis (JIA), including interleukin-1β and -6. Therefore, in this cohort study, we assessed whether KD increases the risk of JIA.

**Methods:**

This study enrolled 7009 patients with and 56 072 individuals without KD in the period 2010–2018 from Taiwan’s National Health Insurance Research Database. On the basis of sex, age, and comorbidities, we executed propensity score matching at the ratio 1:8. The adjusted hazard ratio (aHR) for JIA was determined through multiple Cox regression. Stratified analysis and sensitivity tests were also employed.

**Results:**

When adjusting for age, sex, and comorbidities, the JIA risk was noted to be 2.02-fold greater in children with KD than it was in those without (aHR: 2.02, 95% confidence interval: 1.12–3.67, p = 0.0205). The sensitivity test and subgroup analysis obtained consistent findings in the different sex and comorbidity subgroups.

**Conclusion:**

Children’s risk of JIA is higher if they have KD. Pediatricians should consider the possibility of JIA in this population. More investigations are necessary to identify the pathological mechanisms that link JIA and KD.

## Introduction

Juvenile idiopathic arthritis (JIA) and KD are two distinct systemic inflammatory diseases. They have their own diagnostic criteria but do share numerous clinical features, including such biological markers as interleukin-1β (IL-1β) and IL-6 (1, 2).

For KD, the patients with which are usually children (3–5), intravenous immunoglobulin was discovered to lower the incidence of cardiovascular abnormalities from 25% to <5% (3).

A common complication of KD, arthritis was reported to occur in 30% of patients with this disease in a study conducted before intravenous immunoglobulin was widely used (3). Arthritis is a self-limiting condition and has no association with cartilage destruction (6); several cases of JIA have been reported (7). Diagnosis of KD or JIA is challenging owing to their shared features and the lack of knowledge on relevant biomarkers (7, 8). Kawasaki-disease-related arthritis is more frequently associated with coronary artery abnormalities than is non-Kawasaki-disease-related arthritis (9, 10).

Whether KD increases the risk of JIA in children has not yet been determined. Unfortunately, little evidence has been obtained regarding the correlation between KD and JIA. In addition, no study has yet employed a large national database to longitudinally assess this correlation. KD was hypothesized in this study to increase the JIA risk. Accordingly, Taiwan’s National Health Insurance Research Database (NHIRD) was employed to obtain a real-world, population-based retrospective cohort to test this hypothesis.

## Methods

### Data source

The NHIRD includes the health-care data of approximately 23 million National Health Insurance beneficiaries (>99% of the population). These data are claims data and include records relating to hospitalizations and outpatient and emergency visits. The Longitudinal Health Insurance Database, constituting an NHIRD subset, is composed of data on 1 million National Health Insurance beneficiaries for the period 2010–2018, a sample that was obtained through random sampling of all 23 million beneficiaries. Before researchers are given access to the database, all patient data are deidentified to ensure the patients’ privacy. We executed our current study after receiving ratification from Chung Shan Medical University Hospital’s Institutional Review Board (no. CS19159).

### Cohort and study outcome

The cohort in this study comprised children who received a new diagnosis of KD (11–13) (*International Classification of Diseases, 9th Revision, Clinical Modification* [*ICD-9-CM*] code 446.1 and *ICD-10-CM* code M30.3) from 2010 to 2018. The diagnosis of KD in Taiwan would have been confirmed as American Heart Association diagnosis criteria of KD, which meet one principal diagnosis and up to four secondary diagnoses. Therefore, we exclude the incomplete type or atypical KD patients based on the criteria. To ensure diagnostic accuracy, children with any diagnosis of KD prior to 2010 were excluded from this study. Only children with at least 1 hospitalization were selected for the final analysis.

The non-Kawasaki-disease group contained children without a diagnosis of KD during 2010–2018. We considered the 30-day time point after KD diagnosis as the index date. To ensure that all of the included individuals had a new diagnosis of JIA, we excluded any pre-index-date diagnosis of JIA (*ICD-9-CM* codes 714 and 720; *ICD-10-CM* codes M05, M06, M08, and M45) (14).

A diagnosis of JIA, with the patient being hospitalized at least once or making one or more outpatient visits, was this study’s outcome variable. The follow-up period employed in this study was up to the occurrence of JIA; December 31, 2018; or withdrawal from the National Health Insurance system, whichever occurred first.

### Covariates and matching

The following demographic and medical characteristics were considered in the present study: sex, level of urbanization of residence, age, and related comorbidities—autism (*ICD-9-CM* code 299.0; *ICD-10-CM* code F84.0), attention deficit hyperactivity disorder (*ICD-9-CM* code 314.0; *ICD-10-CM* codes F90, F98.8, and R41.840), asthma (*ICD-9-CM* code 493; *ICD-10-CM* codes J44 and J45), atopic dermatitis (*ICD-9-CM* code 691; *ICD-10-CM* codes L20 and L22), allergic rhinitis (*ICD-9-CM* code 477; *ICD-10-CM* code J30), and Salmonella infection (*ICD-9-CM* code 003; *ICD-10-CM* code A02). A patient was defined as having these comorbidities when they made at least one outpatient visit or had one hospitalization during the 12 months preceding the index date.

The two groups’ heterogeneity was balanced through matching of propensity scores, probabilities that are estimated through logistic regression. The groups were matched on the basis of sex, level of urbanization of residence, age, attention deficit hyperactivity disorder, allergic rhinitis, asthma, and atopic dermatitis. The binary variable was developing versus not developing KD.

### Statistical analysis

The two groups were compared by utilizing absolute standardized differences. An absolute standardized difference < 0.1 indicated that the groups had similar characteristics. To estimate the hazard ratio for JIA in the Kawasaki-disease group versus the non-Kawasaki-disease group, we employed the Cox proportional-hazard model. The Kaplan–Meier method was employed to determine the JIA cumulative incidence; in addition, the log-rank test was next executed to test for significance.

## Results

**Figure 1:**
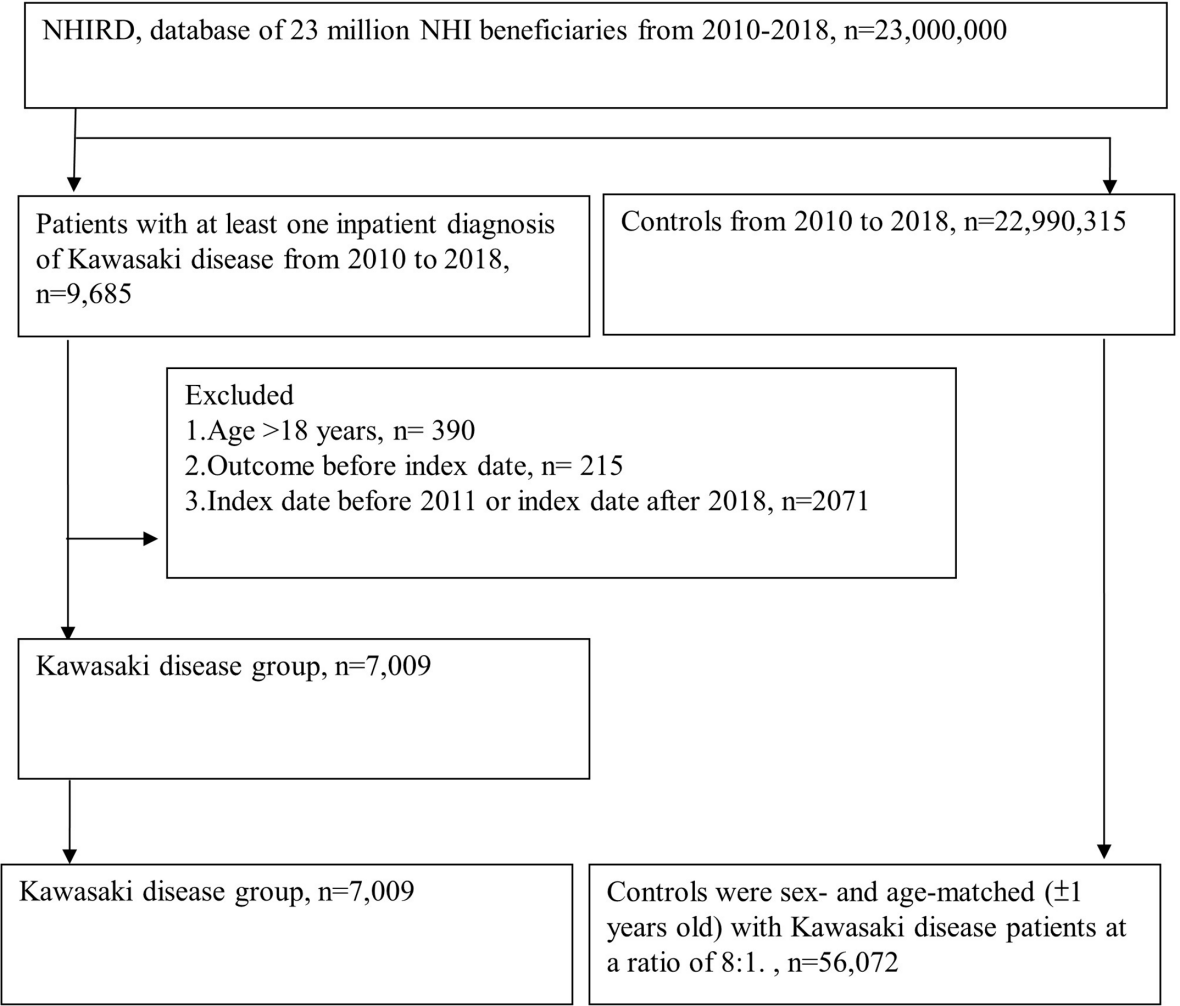
Flowchart detailing the Kawasaki-disease and non-Kawasaki-disease group enrollment.

As displayed in **Figure 1**, from the Longitudinal Health Insurance Database for the period 2010–2018, 7009 patients with KD and 56 072 matched controls could be identified. **Table 1** shows that the sex and age distributions in the two groups were similar.

**Table 1:** Demographic characteristics of the patients with KD and matched controls.

	Control	KD	p-value	ASD
	(N=56072)	(N=7009)		
Index year			1.0000	0.0000
2011,2012,2013	23024 (41.06%)	2878 (41.06%)		
2014,2015	15568 (27.76%)	1946 (27.76%)		
2016,2017	17480 (31.17%)	2185 (31.17%)		
Sex			1.0000	0.0000
Female	22496 (40.12%)	2812 (40.12%)		
Male	33576 (59.88%)	4197 (59.88%)		
Age			0.0043	0.0670
0-3	46070 (82.16%)	5868 (83.72%)		
4-5	5939 (10.59%)	665 (9.49%)		
6-18	4063 (7.25%)	476 (6.79%)		
mean ± SD	2.44 ± 2.15	2.12 ± 2.26		
Urbanization			0.0006	0.0527
Urban	35585 (63.46%)	4567 (65.16%)		
Suburban	17145 (30.58%)	2096 (29.9%)		
Rural	3342 (5.96%)	346 (4.94%)		
Comorbidity				
ADHD	163 (0.29%)	10 (0.14%)	0.0255	0.0318
Autism	59 (0.11%)	10 (0.14%)	0.3712	0.0106
Asthma	2236 (3.99%)	275 (3.92%)	0.7955	0.0033
Atopic dermatitis	4779 (8.52%)	765 (10.91%)	<.0001	0.0808
Allergic rhinitis	3396 (6.06%)	647 (9.23%)	<.0001	0.1197
Salmonella infection	214 (0.38%)	39 (0.56%)	0.0291	0.0256

ASD, absolute standardized difference; ADHD, attention deficit hyperactivity disorder.

The intergroup differences after propensity score matching were discovered to be nonsignificant.

After making adjustments in the multivariate analysis, we discovered the JIA risk to be significantly greater for the children with KD than for those without (adjusted hazard ratio [aHR]: 2.02, 95% confidence interval [CI]: 1.12–3.67, p = 0.0205; **Table 2**). In terms of comorbidities, individuals with allergic rhinitis had a higher JIA risk (aHR: 3.06, 95% CI: 1.60–5.84, p < 0.001; **Table 3**). Children in the Kawasaki-disease group aged 6–18 years had a higher JIA risk than did those aged 0–3 years (aHR: 2.82, 95% CI: 1.49–5.34, p = 0.0015). **Table 4** details the demographic-characteristic-based subgroup analyses of associations between KD and JIA. Those in the Kawasaki-disease group aged 0–3, 4–5, and 6–18 years had a 1.84-fold, 1.89-fold, and 3.1-fold greater risk of JIA, respectively (aHR: 1.84, 95% CI: 0.88–3.83, p = 0.104; aHR: 1.89, 95% CI: 0.20–17.94, p = 0.578; aHR: 3.1, 95% CI: 0.95–10.11, p = 0.061, respectively). The JIA risk was noted to be 3.01-fold greater in girls with KD than it was in girls without KD (aHR: 3.01, 95% CI: 1.38–6.54, p = 0.005), but it was noted to be 1.28-fold greater in boys with KD than it was in boys without KD (aHR: 1.28, 95% CI: 0.49–3.34, p = 0.612). Furthermore, the Kawasaki-disease group had a higher JIA risk regardless of comorbidities or level of urbanization of residence. We also observed the JIA risk to be greater in children with KD diagnosed through catastrophic disease certification than it was in those without KD (aHR: 3.11, 95% CI: 1.21–7.96, p = 0.0181; **Table 5**).

**Table 2:** Incidence of JIA .

	Control	KD	p-value
Follow-up person-months	2899366	361847	
New case	51	14	
Incidence rate†	0.18 (0.13-0.23)	0.39 (0.23-0.65)	
Crude HR (95% CI)	Reference	2.20 (1.22-3.98)	0.0090
Adjusted HR (95% CI)	Reference	2.02 (1.12-3.67)	0.0205

† per 10 000 person-months

aHR, adjusted hazard ratio. Demographic variables (such as age, sex, and level of urbanization), attention deficit hyperactivity disorder, asthma, atopic dermatitis, and allergic rhinitis were included in the multiple Cox regression.

**Table 3:** Hazard ratios for JIA in the multiple Cox regression.

	aHR (95% C.I.)	p-value
KD (ref:control)	2.02 (1.12-3.67)	0.0205
Sex		
Female	reference	
Male	0.64 (0.39-1.04)	0.0695
Age (years)		
0-3	reference	
4-5	0.90 (0.38-2.15)	0.8190
6-18	2.82 (1.49-5.34)	0.0015
Urbanization		
Urban	reference	
Suburban	0.82 (0.44-1.54)	0.5371
Rural	1.34 (0.50-3.59)	0.5559
Comorbidity		
ADHD	2.89 (0.38-21.94)	0.3045
Asthma	0.62 (0.21-1.84)	0.3898
Atopic dermatitis	1.24 (0.58-2.65)	0.5753
Allergic rhinitis	3.06 (1.60-5.84)	0.0007

aHR, adjusted hazard ratio; ADHD, attention deficit hyperactivity disorder.

**Table 4:** Subgroup analysis: Relationship between KD and JIA development.

	N	No. of JIA	Incidence rate†		Adjusted HR (95% CI)	p-value
			Control	KD		
Sex						
Female	25308	32	0.20 (0.13-0.30)	0.63 (0.33-1.22)	3.01 (1.38-6.54)	0.0054
Male	37773	33	0.16 (0.11-0.23)	0.23 (0.09-0.55)	1.28 (0.49-3.34)	0.6118
p for interaction					0.2041	
Age (years)						
0-3	51938	45	0.15 (0.11-0.21)	0.3 (0.16-0.58)	1.84 (0.88-3.83)	0.1040
4-5	6604	6	0.16 (0.07-0.38)	0.28 (0.04-1.99)	1.89 (0.2-17.94)	0.5780
6-18	4539	14	0.43 (0.23-0.8)	1.47 (0.55-3.93)	3.1 (0.95-10.11)	0.0608
p for interaction					0.8238	
Comorbidity						
With Allergic rhinitis	4043	14	0.53 (0.29-0.96)	0.73 (0.24-2.27)	1.34 (0.37-4.85)	0.6580
Without Allergic rhinitis	59038	51	0.15 (0.11-0.2)	0.34 (0.19-0.62)	2.31 (1.18-4.51)	0.0144
p for interaction					0.4101	

† per 10 000 person-months

aHR, adjusted hazard ratio; ADHD, attention deficit hyperactivity disorder. ADHD, asthma, atopic dermatitis, allergic rhinitis, and demographic variables were included in the multiple Cox regression.

**Table 5:** Sensitivity analysis (definition of KD: catastrophic disease): Incidence of JIA.

	Control	KD	p-value
Follow-up person-months	2899366	69929	
New case	51	5	
Incidence rate†	0.18 (0.13-0.23)	0.72 (0.3-1.72)	
Crude HR (95% CI)	Reference	4.06 (1.62-10.19)	0.0028
Adjusted HR (95% CI)	Reference	3.11 (1.21-7.96)	0.0181

† per 10 000 person-months

aHR, adjusted hazard ratio; ADHD, attention deficit hyperactivity disorder. ADHD, atopic dermatitis, asthma, allergic rhinitis, and demographic variables were included in the multiple Cox regression.

**Figure 2:**
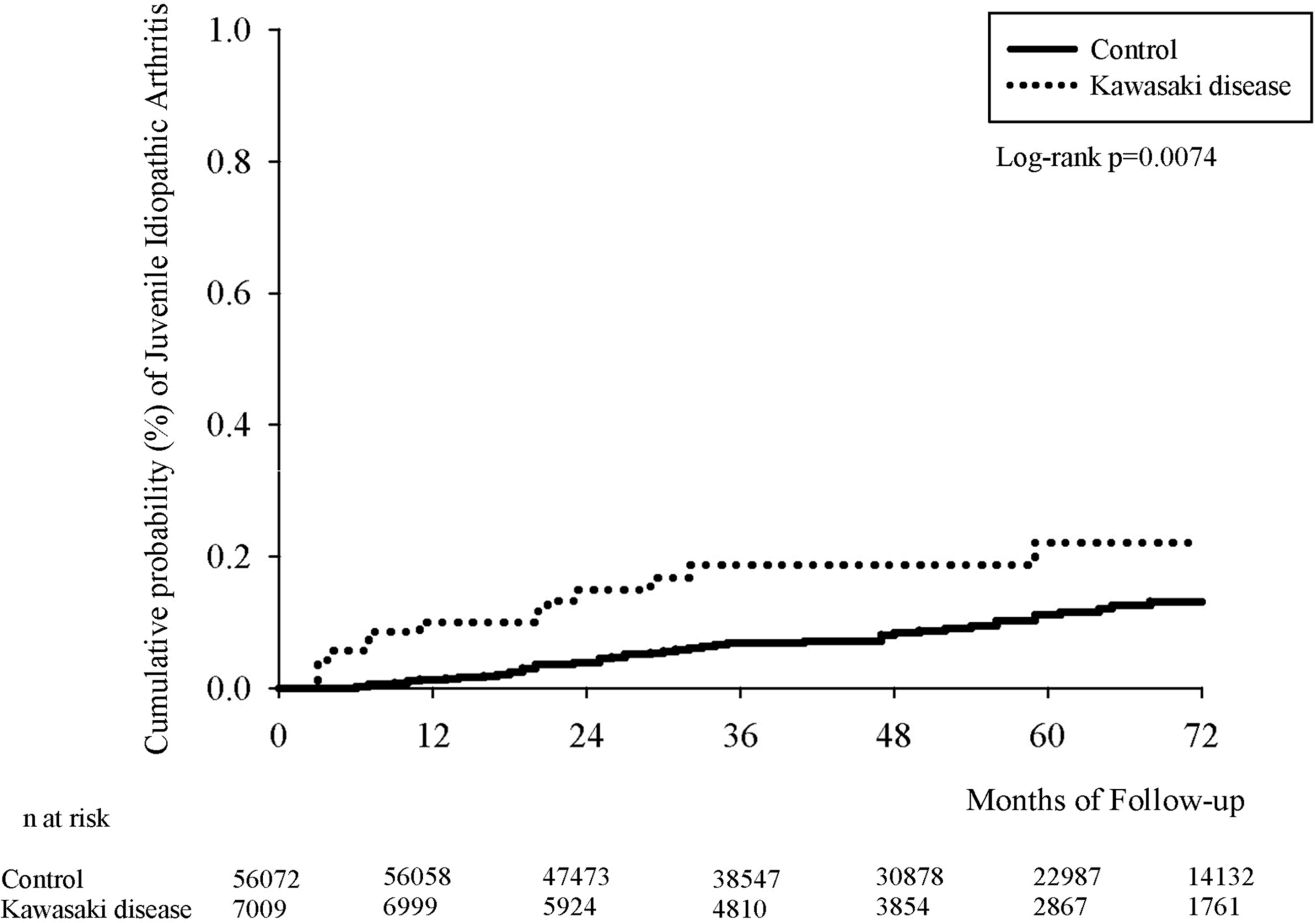
Kaplan–Meier curve of cumulative incidence proportion of JIA in the Kawasaki-disease and non-Kawasaki-disease groups.

**Figure 2** presents the Kaplan–Meier curves for our cohort. We determined the Kawasaki-disease group to exhibit a significantly higher cumulative incidence of JIA when compared with that in the non-Kawasaki-disease group. In the comparison of cumulative incidence curves, the log-rank test returned p = 0.007.

## Discussion

In our current study, children with KD had a 2.02-fold higher JIA risk than did those without KD, regardless of sex, comorbidities, age, or level of urbanization of residence. This is the first research to analyze the association between KD and JIA in the setting of Taiwan. In addition, this appears to be the largest epidemiological study to probe the KD–JIA association by employing a nationwide longitudinal population-based dataset. The risk of JIA should be explained to the parents of children with KD, and these patients should be given appropriate management for JIA as required.

A study conducted recently in developed countries reported the prevalence of JIA to be up to 150 per 100 000 children (15). Because of differences in classification criteria, the studied population, and research methods, the findings related to JIA vary widely (16, 17). The JIA prevalence was 1.33 per 100 000 children in one study (18). The striking association of KD with JIA indicates that these two diseases have common immunobiology and similar cytokine signatures, biologic agents, and immune activation (19–23). In Taiwan, the JIA classification of the International League of Associations for Rheumatology (final revision) is employed to define diagnostic criteria (24).

IL-1β is a mediator of synovial inflammation and is released in response to injury or stress (25). Serum from patients with JIA was discovered to produce IL-1β after being stimulated, and IL-1β was relieved from peripheral blood cells (26). Data obtained in animal and genetic studies of KD indicate that IL-1β is involved in intravenous immune globulin, response to cardiac outcomes, and inflammation (27–29). Serum from patients with KD was found to persistently exhibit elevated IL-6 and IL-8 levels (22). IL-1β blockade in children with JIA may reduce the proportion of children with KD with excessive IL-1β production despite intravenous immunoglobulin therapy. The efficacy of canakinumab (30) and anakinra (31) is the same as that of IL-1 β blockade. In KD, anti-IL-1β treatment may help prevent coronary artery disease, although more studies are required on this topic. In JIA, blockade of IL-6 can reduce inflammation; thus, IL-6 can be concluded to play a major role in this disease (32, 33).

The bloodstream contains inflammatory cytokines, which continually infiltrate endothelial cells and structures by inducing inflammation (34). As KD progresses, C-reactive protein levels increase in tandem with increases in IL-1β and IL-6 Levels (35, 36). In 1991, Newburger proposed intravenous immunoglobulin therapy, which has become the conventional treatment for KD (37, 38). Although the mechanism behind this treatment remains unclear, intravenous immunoglobulin is thought to reduce the levels of inflammatory cytokines and thus achieve their balance. Intravenous immunoglobulin was reported by a previous study to reduce the level of IL-6 to within the normal range, although that study also noted the soluble IL-6 receptor and TNF-α levels to be unaffected by this therapy (39). Researchers have discovered that in KD’s pathogenesis, one critical factor is the presence in the blood of inflammatory cytokines (40, 41).

KD and JIA each have their own diagnostic criteria but initially have similar clinical manifestations. In one study, 0.5% of children with KD had JIA, whereas KD preceded JIA in 7% of cases (1). This percentage is higher than that reported in a study published in 2015, which revealed the KD–JIA incidence to be 0.2% among 6745 patients with KD, the data of whom were derived from a database containing patients’ health information (7). Patients satisfying both the KD and JIA diagnostic criteria were reported to exhibit a lower albumin level and higher leukocyte count and to be more refractory to standard intravenous immunoglobulin therapy (1). Arthritis was found in 7.5% of patients with KD, further highlighting the clinical similarity of these two diseases (42). The many clinical features shared by JIA and KD appear to be more persistent in JIA and more self-limiting in KD (1).

This study does have some limitations. First, in the NHIRD, KD and JIA diagnoses are both based on *ICD-9-CM* and *ICD-10-CM* codes. Therefore, our current study may have underestimated the incidence of JIA because patients who had mild symptoms may not have been hospitalized or brought to the outpatient department. Second, it was not possible to further analyze and discuss the results for JIA subtypes because the dataset did not contain related information. Third, the NHIRD does contain data on covariates, such as laboratory findings, environmental factors, social adversity, genetics, personal lifestyles, and family history. Although we performed propensity score matching and adjusted for various possible confounders, such as lifestyle-related comorbidities, unmeasured confounding factors may have influenced the present findings. Finally, considering that we derived our results from analyzing Taiwanese patient data, it may not be possible to directly generalize our findings to other ethnic groups. Scholars should conduct studies involving other nationalities and ethnicities to determine how generalizable is the relationship discovered in this study.

In conclusion, children with KD were at a 2.02-fold higher JIA risk than children without KD, regardless of sex, level of urbanization, age, or comorbidities. Therefore, when caring for a child with KD, the clinician should be aware of the possibility that other systemic inflammatory disorders, such as JIA, can develop in the future. The pathophysiological association between KD and JIA requires further study.

## Data availability statement

The original contributions presented in the study are included in the article/supplementary material. Further inquiries can be directed to the corresponding authors.

## Author contributions

L-CL and Y-HF drafted the manuscript. JW revised the manuscript critically. All authors contributed to the article and approved the submitted version.
